# Suggested Standards of Purity for Foods and Drugs

**Published:** 1903-03

**Authors:** 


**Suggested Standards of Purity for Foods and Drugs.** By C. G. Moor. Pp. 260. London: Baillière, Tindall & Cox. 1902.

This work deals chiefly with the drugs and galenical preparations of the *British Pharmacopœia*, and to a slight extent only with articles of food.

The present time is opportune for the publication of these suggested standards of purity for drugs, when the question is receiving considerable attention from medical men and pharmacists throughout the country.

The author summarises the analytical data of numerous workers upon official drugs and galenical preparations. In the case of drugs owing their therapeutic activity to definite substances which can be determined by chemical methods, standardisation is readily attainable, and, in many important instances, was adopted in the *British Pharmacopœia* of 1898.

Extension of the principle to other important official drugs is advocated, and standards of active principles suggested.

In the many instances where no definite active constituents can be isolated, the author, following the course adopted by the *British Pharmacopœia*, suggests percentage of extractive matter yielded to certain solvents, percentage of ashes, and limits of water, as indicating standard quality.

Galenical preparations of the nature of tinctures, liquid extracts, etc., are to be examined with regard to specific gravity, percentage of total solids, and percentage of alcohol. In addition, determination of active principles is to be made wherever practicable.

The author quotes extensive data, showing what limits may reasonably be expected in genuine preparations.

The standards to be aimed at in the case of crude drugs are: attainment of a definite quality, and freedom from admixture and sophistication. In the case of preparations of drugs, an approximately constant therapeutic value is demanded, as indicated by active principles, or where these cannot be determined, by agreement with standard constants.

We notice that in dealing with ipecacuanha, the author states that there is no means of ascertaining whether the official or non-official root has been used in preparing the liquid extract or wine. He overlooks the fact that the relative proportions of the two chief alkaloids differ considerably in the Rio and Carthagena roots, and that as the ratio of emetine to cephæline in each root is approximately constant, the source of the preparation may be determined by a rough separation of the total mixed alkaloids obtained.

The question of physiological standardisation is left untouched.

